# Methotrexate treatment of FraX fibroblasts results in *FMR1* transcription but not in detectable FMR1 protein levels

**DOI:** 10.1186/1866-1955-5-23

**Published:** 2013-09-10

**Authors:** Cornelia Brendel, Benjamin Mielke, Merle Hillebrand, Jutta Gärtner, Peter Huppke

**Affiliations:** 1 Department of Pediatrics and Pediatric Neurology, Georg August University, Robert-Koch-Strasse 40, 37075 Göttingen, Germany; 2 Department of Anesthetics, Georg August University, Robert-Koch-Strasse 40, 37075 Göttingen, Germany; 3 Department of Pediatrics and Pediatric Neurology, University Medical Center Göttingen, Georg August University, Robert-Koch-Strasse 40, 37075 Göttingen, Germany

## Abstract

**Background:**

Fragile X syndrome is caused by the loss of FMRP expression due to methylation of the *FMR1* promoter. Treatment of fragile X syndrome patients’ lymphoblastoid cells with 5-azadeoxycytidine results in demethylation of the promoter and reactivation of the gene. The aim of the study was to analyze if methotrexate, an agent which also reduces DNA methylation but with less toxicity than 5-azadeoxycytidine, has therapeutic potential in fragile X syndrome.

**Methods:**

Fibroblasts of fragile X syndrome patients were treated with methotrexate in concentrations ranging from 1 to 4 μg/ml for up to 14 days. *FMR1* and FMRP expression were analyzed by quantitative PCR and western blotting.

**Results:**

*FMR1* mRNA was detected and levels correlated positively with methotrexate concentrations and time of treatment, but western blotting did not show detectable FMRP levels.

**Conclusions:**

We show that it is possible to reactivate *FMR1* transcription in fibroblasts of fragile X syndrome patients by treatment with methotrexate. However, we were not able to show FMRP expression, possibly due to the reduced translation efficacy caused by the triplet repeat extension. Unless *FMR1* reactivation is more effective *in vivo* our results indicate that methotrexate has no role in the treatment of fragile X syndrome.

## Background

Fragile X syndrome (FXS, OMIM #300624) is the most common monogenic cause of mental retardation. It is caused by the expansion of a polymorphic CGG triplet repeat in the 5′-untranslated region (UTR) of the fragile X mental retardation 1 (*FMR1*) gene located on the long arm of the X-chromosome (Xq27.3) [1]. In the normal population the repeat size is 6 to 55; individuals with a repeat size of 55 to 200 are considered permutation carriers and in FXS patients repeats of >200 are found (full mutation) [2,3]. The full mutation leads to the loss of a DNA-methylation boundary 650 to 800 nucleotides upstream of the CGG repeat in the first exon of the *FMR1* gene [4]. In healthy individuals this boundary protects the unmethylated *FMR1* promoter from spreading methylation. In FXS the promoter is methylated, leading to silencing of the *FMR1* gene resulting in absence of the fragile X mental retardation protein (FMRP) [5,6]. The hypothesis that the FXS phenotype is caused by the loss of FMRP is supported by the description of FXS patients with point mutations in the *FMR1* gene that lead to the production of a nonfunctional protein [7,8]. Intriguingly, rare male individuals have been described who carry a full mutation allele but have normal intelligence, and, in these individuals, the *FMR1* promoter has been found unmethylated and FMRP expressed [9-12]. These findings have stimulated experiments that aimed to reactivate the *FMR1* gene by inducing demethylation. Chiurazzi *et al*. were able to show that *in vitro* treatment of fragile X patients’ lymphoblastoid cells with 5-azadeoxycytidine (5-azadC) leads to expression of *FMR1* mRNA and FMRP [13]. The promoter of the *FMR1* gene was unmethylated after 5-azadC treatment [14]. Unfortunately, 5-azacytidine, a cytosine analog that inhibits DNA methyltransferases and was developed for cancer therapy, is very toxic, thus making treatment of FXS patients not feasible [15]. Here we show that treatment with methotrexate (MTX), an agent used for the long-term treatment of patients with rheumatoid arthritis and found to decrease cellular methylation including DNA methylation, also results in *FMR1* expression but no detectable FMRP levels [16-18].

## Methods

### Cell culture and drug treatment

Human fibroblasts were purchased from the Coriell Institute (Camden, New Jersey, USA) and maintained as monolayer cultures in Dulbecco’s modified Eagle’s medium (DMEM/low glucose, PAA Laboratories GmbH, Pasching, Austria) supplemented with 5 to 10% fetal bovine serum (Biochrom AG, Berlin, Germany) and 0.1% L-glutamine (PAA Laboratories GmbH, Pasching, Austria). Cells were incubated at 37°C in an atmosphere of 5% CO_2_. Before treatment, cells were seeded at an initial equal concentration in a total volume of 10 ml per culture plate and the amount of fetal bovine serum in the culture medium was reduced to 5%. Cells were treated daily with 0.5 to 4 μg/ml methotrexate (Calbiochem, Billerica, MA, USA) and 1 μg/ml 5-aza-2′-deoxycytidine (Sigma, Deisenberg, Germany), respectively. All treatments were performed in triplicate. During treatment, the culture medium was changed every 48 hours. Control plates included FXS patient fibroblasts without treatment, and fibroblasts from a healthy male. After 4 to 14 days all cells were harvested and RNA was isolated.

### RNA extraction

Cell pellets for the extraction of RNA were prepared from each treatment by trypsinization. Total RNA was isolated using peqGOLD TriFast reagent (PEQLAB Biotechnologie GMBH, Erlangen, Germany) and QIAamp™ DNA Kits (QIAGEN, Hilden, Germany), as recommended by the manufacturer. RNA concentrations were measured using a UV spectrophotometer (Nano Drop ND-1000, Thermo Fisher Scientific, Wilmington, DE, USA).

### Reverse transcription PCR

For cDNA synthesis, 2 to 3 μg of total RNA were reverse transcribed (RT) in a 20 μl volume using oligo(dT)_15_ primers and SuperScript™ III Reverse Transcriptase (SuperScript™ III First-Strand Synthesis System, Invitrogen, Karlsruhe, Germany) according to manufacturer’s recommendation. Transcription amplification was performed using the forward primer 5′ gctaaagtgaggatgataaag 3′ and the reverse primer 5′ atccttatgtgccgcctctttgg 3′ producing a 204-bp fragment. As internal control of amplification, oligonucleotides GAPDH forward (5′ gagtcaacggatttggtcgt 3′) and GAPDH reverse (5′ gacaagcttcccgttctcag 3′) were employed to amplify a specific 185-bp product of the *GAPDH* gene. Thirty PCR cycles (95°C, 30 seconds; 57°C, 30 seconds; and 72°C, 40 seconds) were done using HotStar-Taq polymerase with Q-Solution (Qiagen, Hilden, Germany). The amplification products were analyzed on 2% agarose gels supplemented with GelRed (Biotrend, Cologne, Germany).

### Quantitative real-time PCR

First strand cDNAs for quantitative real-time PCR (qRT-PCR) of RNA generated from treated, untreated and control fibroblasts were synthesized as described above. Quantitative RT-PCR was performed using the iQ5 cycler (BioRad Laboratories, Munich, Germany) and the iQ SYBR Green Supermix kit (BioRad Laboratories, Munich, Germany). All qRT-PCR reactions were performed in triplicate. Annealing temperatures for the genes were set to 57°C. Reaction specificity was controlled by post-amplification melting curve analysis. Relative expression rates of target gene transcripts were calculated using the 2^-(ΔΔCt)^ method.

### Methylation analysis

Fibroblasts were treated for 7 days with either 1 μg/ml 5-aza-2′-deoxycytidine or MTX 2 μg/ml. Methylation status of the *FMR1* promoter was analyzed by bisulfite conversion of unmethylated cytosines using the using EpiTect Bisulfite Kit (Qiagen, Venlo, Netherlands). Oligonucleotides were designed as described by Pascale *et al*. [19].

### Western blot analysis

5azadC and MTX-treated and untreated human fibroblasts were washed with PBS. Then cells were lysed in 750 μl RIPA buffer (150 mM NaCl, 50 mM Tris–HCl pH 7.5, 0.1% SDS, 0.5% sodium deoxycholate, 1% NP-40, protease inhibitors) per 10-cm plate for 15 minutes at 4°C. Lysates were clarified by centrifugation (18000×g, 20 minutes at 4°C). A total of 50 μg of protein lysate were separated on 10% SDS-PAGE and transferred to nitrocellulose membrane for 90 minutes with 2mA/cm^2^ using blotting buffer (48 mM Tris–HCl, 39 mM glycine, 0.04% w/v SDS, 20% methanol). Membranes were blocked in PBST + 5% defatted milk followed by overnight incubation at 4°C with a monoclonal antibody against FMRP (Euromedex Souffelweyersheim, Cedex, France). Incubation with appropriate secondary HRP-labelled antibody was followed by detection with Lumi-Light Western blotting substrate (Roche, Mannheim, Germany).

## Results

**Figure 1:**
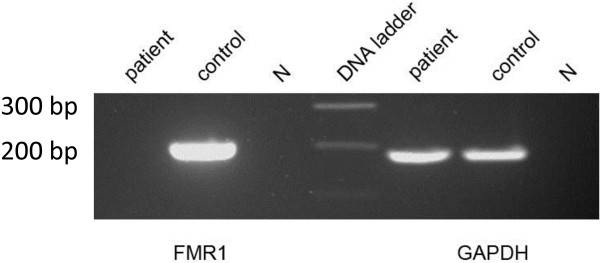
***FMR1 *transcription in fragile X syndrome (FXS) patient and control fibroblasts.** cDNA was synthesized from FXS patient fibroblasts and control fibroblasts followed by PCR. A total of 5 μl of PCR products for *FMR1* and 3 μl of PCR products for GAPDH were analyzed on a 2% agarose gel.

RNA was isolated from fibroblast cell lines from a male FXS patient and a healthy male and used for cDNA synthesis. With oligonucleotides binding in exon 4 and exon 6 of the *FMR1* gene, a 204-bp fragment of *FMR1* was amplified. As expected, no *FMR1* transcript was detected in the patient’s cells (Figure 1).

**Figure 2:**
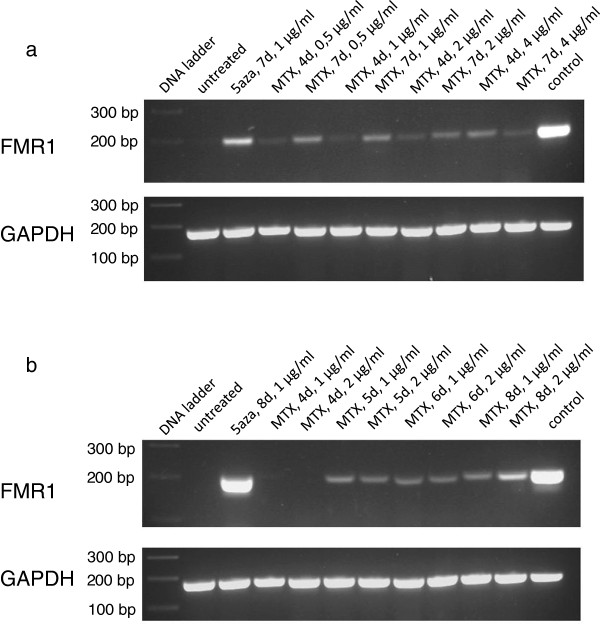
**Effect of methotrexate (MTX)-treatment time and dosage on restoration of *FMR1 *transcription.** Patient fibroblasts were treated with MTX and cDNAs were synthesized followed by PCRs. PCRs were analyzed on 2% agarose gels. The upper panels of each figure show analysis of 5 μl FMR1-PCRs, the lower panels show analysis of 3 μl GAPDH-PCRs. **a)** Cells were treated 4 and 7 days with 0.5, 1, 2 and 4 μg MTX per ml cell culture medium. **b)** Cells were treated 4, 5, 6 and 8 days with 1 and 2 μg MTX per ml cell culture medium.

Since previous work showed that treatment with 5-aza-2′-deoxycytidine (5-azadC) could restore transcription of the *FMR1* gene [13], this substance was included as positive control. The fibroblasts were treated with varying time spans and concentrations of MTX in the culture medium. The experiments showed that it is possible to restore low levels of *FMR1* expression in fibroblasts of the FXS patient. The minimal time span was 4 days (data not shown). The signal of the bands increased with an increasing time span of the treatment and was stronger when the medium contained 2 μg per ml MTX compared to 1 and 0.5 μg/ml. Using 4 μg MTX/ml did not seems to enhance the effect any further (Figure 2a and 2b).

**Figure 3:**
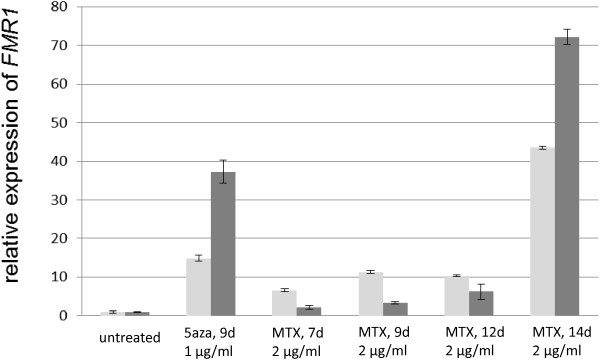
**Quantitative analysis of the methotrexate (MTX) effect on fragile X syndrome (FXS) patient fibroblasts.** Patient fibroblasts were treated with 2 μg MTX per ml culture medium for 7, 9, 12 and 14 days. cDNAs were synthesized and real-time PCRs were performed. The relative *FMR1*-expression of the treated cells is illustrated. The light and the dark grey bars show the results of two separate experiments.

To quantify the effect of MTX treatment on *FMR1* gene transcription, we performed real-time PCRs. Cells were treated for 7 to 14 days with 2 μg MTX per ml cell culture medium (Figure 3). As seen in the previous experiments, restoration of *FMR1* expression increased with the time of MTX exposure. Surprisingly there was a steep rise in expression between day 12 and 14. The experiment was repeated and again the result showed a similar pattern.

**Figure 4:**
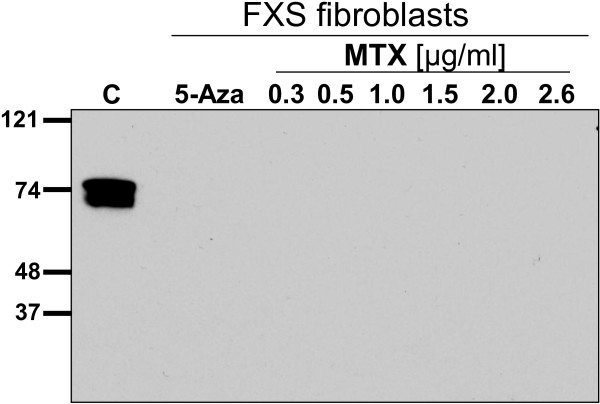
**Western blot analysis after treatment with methotrexate (MTX).** Patient fibroblasts were treated with varying concentration of MTX or 5-azadeoxycytidine (5-azadC) 1 μg/ml for 9 days. C = control fibroblasts.

Western blot was used to analyze FMR1 protein (FMRP) expression. However, we were not able to show reliably any FMRP expression in the patient’s cells treated with either MTX or 5-aza-2′-deoxycytidine. In the fibroblast of the healthy control a very robust signal for FMRP was detected (Figure 4).

**Figure 5:**
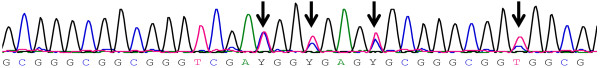
**Analysis of cytosine methylation in the *FMR1 *promoter after treatment with 5-azadeoxycytidine (5-azadC).** The arrows indicate CpGs that were partially demethylated.

Analysis of the methylation of the *FMR1* promoter after treatment with MTX did not demonstrate demethylation while it showed a variable degree of demethylation after treatment with 5-aza-2′-deoxycytidine (Figure 5).

## Discussion and conclusions

Currently several clinical studies addressing various aspects of FXS are underway (clinicaltrials.gov). Most of these studies are based on the 'mGluR' theory formulated in 2004 by Bear, Huber and Warren [20]. However, FMRP is implicated in various aspects of activity dependent mRNA metabolism, and it has been found to interact with 4% of mRNAs in human fetal brain [21,22]. A therapy directed to downstream targets of FMRP is therefore likely to correct only some of several aspects of the FXS phenotype. Reactivation of the gene, on the other hand, would offer a possibility to correct all aspects. Studies in unaffected males with a full mutation who were found to have reduced FMRP levels indicate that the reactivation does not need to be complete [10,11]. Furthermore, the description of variable FXS phenotypes within one family due to variable degrees of methylation might indicate that the non-physiological methylation of the *FMR1* gene promoter is less stable than in physiologically methylated promoters, possibly facilitating reactivation without disturbing the methylation of other promoters [23]. Using 5-azadC it has been shown that it is possible to reactivate the *FMR1* gene by demethylation of the promoter [13]. We aimed to find a drug that reactivates the *FMR1* gene but with less toxicity than 5-azadC. DNA methylation is mediated by CpG methyltransferases (DNMT1) using S-adenosylmethionine (SAM) as a methyl group donor. Folate in the form of 5-methyltetrahydrofolate is essential for the recycling of SAM after the methylation reaction. Folate deficient diet has been found to cause reduced DNA methylation in humans [24,25]. Methotrexate (MTX) is a folate antagonist that acts by inhibiting dihydrofolate reductase (DHFR), an enzyme that catalyzes the conversion of dihydrofolate to tetrahydrofolate. MTX is used in the long-term treatment of rheumatoid arthritis in children and adults. It has been described that treatment with MTX decreases cellular methylation [16-18]. We therefore decided to study MTX as a potential drug for the reactivation of the *FMR1* gene. Firstly we analyzed *FMR1* mRNA expression in fibroblasts from a male FXS patient and from a healthy control. As expected, we did not detect *FMR1* mRNA in the FXS patients’ fibroblasts. Next, we treated the fibroblasts with MTX and used 5-azadC treatment as a positive control. We found that not only treatment with 5-azadC but also treatment with MTX resulted in detectable *FMR1* mRNA levels. The effect of the MTX treatment was positively correlated with the time of treatment and the dosage of MTX. The results that we obtained from the 5-azadC treatment on *FMR1* mRNA levels were comparable to those reported by Chiurazzi *et al*. [13]. However in contrast to them, when we used western blotting we were not able to detect any FMRP, either in the 5-azadC-treated cells or in the MTX-treated cells. Most likely, the protein levels were too low for detection. It has been described in a lymphoblastoid cell line as well as in a mouse model with an unmethylated full mutation that the CGG repeat itself causes mRNA translation to be 40% less efficient [14,26]. The FMRP levels might therefore be very low. On the other hand, our experiments might understate the effects of MTX *in vivo* because cells grown in culture have been shown to have an excess of *de novo* methylation of CpG islands [27,28]. In fact, we found that our reactivation experiments were less efficient after the fibroblasts had undergone more cell cycles (data not shown).

MTX affects cell metabolism in many ways. It reduces the synthesis of thymidylates and purines as well as the conversion of homocysteine to methionine. MTX treatment thereby leads to impaired DNA and protein synthesis as well as reduced methylation of DNA and proteins. Consequently, at this point of time, we cannot prove that the expression of *FMR1* found after MTX treatment is caused by reduced DNA methylation. In fact DNA methylation after treatment with MTX was not altered. Additionally, the lack of FMR1 protein expression might be caused by the overall reduced protein synthesis. Further experiments directed at understanding the mode of action of MTX in the FXS fibroblasts might also help us to improve the outcome of MTX treatment.

Unfortunately, no mouse model is available for *in vivo* testing because even in mice carrying very long CGG repeats no abnormal methylation occurs [26]. Another opportunity to study the effect of MTX in FXS would be an individual with the comorbidity of FXS and rheumatoid arthritis receiving MTX. Studying *FMR1* mRNA and FMRP expression in lymphocytes of such an individual would help to clarify the potential of MTX in FXS. An inquiry was sent to the German FXS support organization (http://www.frax.de), but no individual in Germany with such comorbidity is currently known. However, due to the high prevalence of FXS and rheumatoid arthritis, it is likely that such an individual exists in another country.

## Abbreviations

FXS: Fragile X syndrome; MTX: Methotrexate; qRT-PCR: Quantitative real-time polymerase chain reaction; RT-PCR: Reverse transcriptase polymerase chain reaction; UTR: Untranslated region; 5-azadC: 5-azadeoxycytidine.

## Competing interests

The authors declare that they have no competing interests.

## Authors’ contributions

CB, BJ carried out the cell culture and drug treatment, the molecular genetic studies, the methylation studies and the western blot experiments. MH carried out the cell culture and drug treatment and the western blot experiments. JG and PH designed the experiments and drafted the manuscript. All authors read and approved the final manuscript.
